# Precision microbiome medicine and therapeutics: the enabling role of *in vitro* gut models

**DOI:** 10.1093/femsre/fuag038

**Published:** 2026-08-03

**Authors:** Zhan Huang, Siew C Ng, Harry Sokol, Nathalie Rolhion

**Affiliations:** Sorbonne Université, Inserm, Centre de Recherche Saint-Antoine, CRSA, F-75012 Paris, France; Gut, Liver & Microbiome Research (GLIMMER) FHU, F-75012 Paris, France; Microbiota I-Center (MagIC), Hong Kong SAR, China; Department of Medicine and Therapeutics, State Key Laboratory of Digestive Diseases, Li Ka Shing Institute of Health Sciences, The Chinese University of Hong Kong, Hong Kong, China; Microbiota I-Center (MagIC), Hong Kong SAR, China; Department of Medicine and Therapeutics, State Key Laboratory of Digestive Diseases, Li Ka Shing Institute of Health Sciences, The Chinese University of Hong Kong, Hong Kong, China; New Cornerstone Science Laboratory, The Chinese University of Hong Kong, Hong Kong, China; Sorbonne Université, Inserm, Centre de Recherche Saint-Antoine, CRSA, F-75012 Paris, France; Gut, Liver & Microbiome Research (GLIMMER) FHU, F-75012 Paris, France; Department of Gastroenterology, Sorbonne Université, AP-HP, Hôpital Saint Antoine, F-75012 Paris, France; INRAE UMR1319 Micalis, AgroParisTech, F-78322 Jouy-en-Josas, France; Sorbonne Université, Inserm, Centre de Recherche Saint-Antoine, CRSA, F-75012 Paris, France; Gut, Liver & Microbiome Research (GLIMMER) FHU, F-75012 Paris, France

**Keywords:** gut microbiome, personalized medicine, *in vitro* human gut microbiota culture, static microbiota model, dynamic microbiota model, microbe-host interaction model

## Abstract

The human gut microbiome exhibits profound interindividual variability, challenging the efficacy of generalized interventions and underscoring the need for precision approaches to microbiome-associated diseases. Such strategies require comprehensive, individualized assessment of microbial ecosystems and their functional responses, an objective that remains difficult to achieve *in vivo. In vitro* gut models offer a promising alternative, maintaining personalized microbial communities in controlled environments and enabling detailed investigation of microbiome dynamics. Complementary microbe–host co-culture systems incorporating human cells further permit mechanistic interrogation of host responses to microbial stimuli. Together, these integrated platforms offer a translational framework for developing tailored therapeutics. In this Perspective, we examine how *in vitro* gut models can advance microbiome-based personalized medicine, discuss key determinants of therapeutic variability, and outline the relevance, limitations, and future directions of these systems in designing targeted, more effective interventions.

## Teaser

This perspective highlights how *in vitro* gut models, ranging from static and dynamic systems to microbe-host co-culture platforms, provide a critical tool for addressing individual microbiome variability and bridging the translational gap toward microbiome-based personalized medicine.

## Introduction

The gut microbiome, with its profound biological significance and remarkable plasticity, underpins a broad spectrum of therapeutic strategies that dynamically shape host immunity, metabolism, and disease trajectories (Sorbara and Pamer 2022). Extensive data demonstrate that this microbial ecosystem contributes to inter-individual variations in treatment response through highly person-specific signatures (Ratiner et al. 2024). As modern medicine advances toward more precise intervention strategies (Jameson and Longo 2015), understanding and harnessing interindividual microbiome variability has become imperative. Microbiome-based personalized medicine thus represents a critical frontier for optimizing therapeutic efficacy and mitigating the burden of complex diseases driven by gut microbial dysbiosis.

A critical first step in advancing microbiome-based personalized medicine is the systematic acquisition of comprehensive, individual-level microbiome data. Although *in vivo* studies are essential, they are limited by high costs, time intensity, ethical constraints, and complex host interactions that complicate mechanistic interpretation and reproducibility (Arrieta et al. 2016, Wilde et al. 2024, Turjeman et al. 2025). In this context, *in vitro* gut models, spanning simple batch fermenters, continuous bioreactors, and gut-on-a-chip systems (Vrancken et al. 2019, Özkan et al. 2024), have emerged as indispensable complementary tools for unraveling the complexities of microbiome-based therapeutics. By enabling controlled, precise, and mechanistic studies under defined conditions, these systems offer a scalable platform for preclinical assessment in microbiome-based personalized medicine.

*In vitro* gut models can effectively replicate individual microbiome profiles and retain personalized responses to various interventions (Li et al. 2019, Aranda-Díaz et al. 2022, Quinn-Bohmann et al. 2024, Rytter et al. 2025). Batch fermentation systems, termed static models, enable high-throughput screening of microbiome-modulating agents (e.g. prebiotics, probiotics, and postbiotics), while dynamic models, including continuous bioreactors and multi-stage systems, better simulate temporal microbial interactions, community stability, and longitudinal responses over extended periods. Complementing these microbiota-focused systems, microbe-host interaction models facilitate detailed mechanistic investigation of host responses to microbial stimuli, providing a robust framework for elucidating personalized microbiome–host dynamics (Ingber 2022). By prioritizing causality over correlation, these strategies optimize personalized microbiome interventions for complex diseases. However, studies systematically applying these models to personalized therapeutic development remain scarce. In this Perspective, we examine the role of person-specific microbial signatures in shaping personalized medicine and analyze key factors that influence responses to microbiome-based therapeutics, with the aim of clarifying the basis of interindividual variability in treatment outcomes. We then evaluate *in vitro* approaches in microbiome research, highlighting how diverse model systems contribute to the advancement of precision strategies. Finally, we discuss current limitations and outline future directions to enhance the translational relevance and predictive power of *in vitro* gut models.

## The gut microbiome in personalized medicine

Medicine is moving from one-size-fits-all approaches to more precise, effective, and patient-centered healthcare, known as personalized medicine. This shift is particularly evident in microbiome-associated diseases, such as metabolic disorders (Fan and Pedersen 2021) and inflammatory diseases (Lavelle and Sokol 2020). The human gut microbiome, a highly plastic and dynamic ecosystem, has emerged as a central mediator in this transition (Schupack et al. 2022). Through its profound influence on host immunity, metabolism, drug disposition, and systemic physiology (Altay et al. 2019, Yamada and Palm 2025, Al-Btoosh et al. 2026), as well as its distinctive person-specific compositional and functional signatures (Lloyd-Price et al. 2017), the gut microbiome drives substantial inter-individual variability in disease susceptibility and treatment responses. Consequently, integrating microbiome profiling into clinical decision-making has become an essential strategy for advancing individualized therapeutic interventions and improving outcomes in complex disorders.

### Person-specific microbial signatures

**Figure 1:**
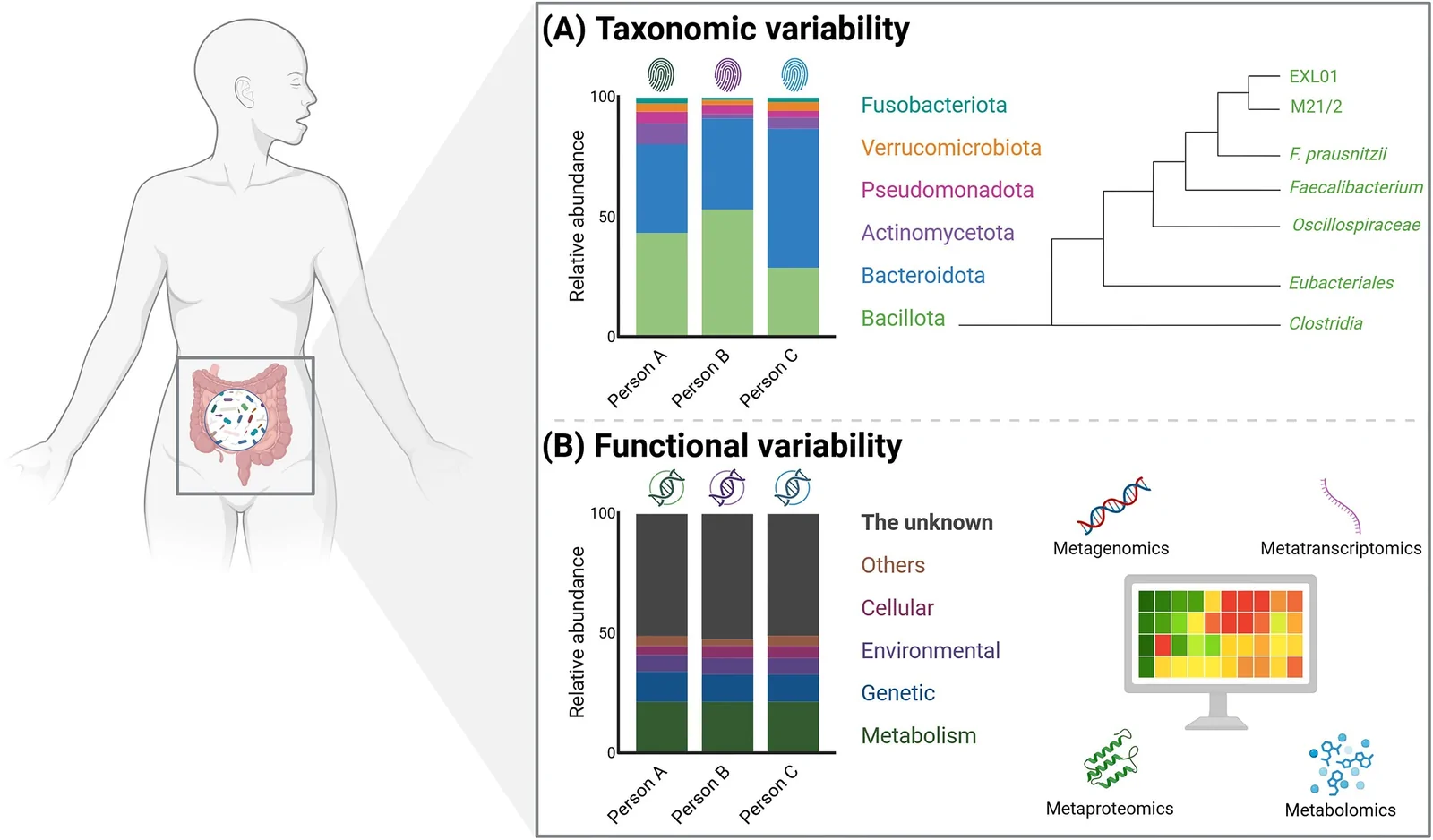
Inter-individual variability in the gut microbiome. (A) Taxonomic variability: The human gut microbiota is characterized by high beta-diversity, where individual-specific signatures are defined by variations in the relative abundance of core phyla and discrete strain-level compositions. This taxonomic fingerprint represents a distinctive microbiota signature unique to each host, shaped by both host and environmental factors. (B) Functional variability: In contrast to taxonomic variability, the metagenomic potential exhibits high conservation across individuals, a phenomenon due to functional redundancy. However, a significant fraction of this collective gene repertoire remains uncharacterized, representing the functional dark matter that constitutes a major knowledge gap in microbial function. While genomic potential remains relatively stable, the realized functional output comprised the active transcriptome, proteome, and metabolome shows heightened inter-individual sensitivity and variability. This is driven by localized gut physiology, dietary intake, and transient environmental perturbations, resulting in highly individualized metabolic phenotypes.

The human gut microbiome comprises trillions of microorganisms and their associated genetic material, serving as a dynamic interface between host physiology and environmental exposures. Bacteria belonging to the phyla Bacillota (formerly Firmicutes) and Bacteroidota (formerly Bacteroidetes) dominate this ecosystem, each represented by numerous genera (Lozupone et al. 2012). Additional phyla, including Actinomycetota (formerly Actinobacteria), Pseudomonadota (formerly Proteobacteria), Verrucomicrobiota, and Fusobacteriota, are also consistently present but at lower relative abundances. At a finer resolution, composition varies markedly at the species level and particularly at the strain level, where intraspecies genetic and metabolic differences give rise to a highly personalized microbial fingerprint that remains relatively stable over time in healthy adults (Lloyd-Price et al. 2017, Chen-Liaw et al. 2025). These layered taxonomic differences collectively define a distinctive microbiota signature unique to each individual host (Fig. 1A).

Beyond taxonomy, shotgun metagenomic profiling reveals pronounced person-specific patterns in the collective gene repertoire (metagenome), including thousands of genes involved in carbohydrate metabolism, vitamin biosynthesis, and xenobiotic degradation (Tierney et al. 2019, Meawad et al. 2025). Although functional redundancy is a defining feature of the microbiome, allowing phylogenetically distinct taxa to fulfill overlapping core metabolic functions and sustain the production of key health-associated metabolites (Moya and Ferrer 2016), the precise composition and concentrations of these metabolites vary substantially between individuals (Treuren Van and Dodd 2020) (Fig. 1B). These variations are largely driven by inter-individual differences in gut physiology and the local intestinal environment (Procházková et al. 2024). Moreover, functional omics approaches (metatranscriptomics, metaproteomics, and metabolomics) demonstrate that the realized functional output exhibits greater inter-individual variability and heightened sensitivity to perturbation than either taxonomic composition or metagenomic potential alone (Heintz-Buschart and Wilmes 2018). Despite advances in high-throughput sequencing and annotation technologies, a substantial proportion of microbial genes remain uncharacterized, underscoring the persistent functional dark matter within the gut ecosystem (Heintz-Buschart and Wilmes 2018, Pavlopoulos et al. 2023). Further work is required to fully interpret the functional potential of the gut microbiome and of its individual members.

Person-specific microbial signatures offer substantial translational potential. Gut microbiome composition has been shown to accurately predict individualized postprandial glycemic responses to real-life meals (Zeevi et al. 2015). A recent large-scale study involving over 10 000 individuals has further demonstrated that diet strongly predicts species-level microbiome composition and can be used to simulate personalized dietary interventions associated with cardiometabolic improvements (Segev et al. 2026). Similarly, systematic mapping has revealed extensive person-specific drug metabolism by the gut microbiome, as demonstrated by quantitative *ex vivo* frameworks linking individual microbial communities to differential drug biotransformation (Javdan et al. 2020). However, the links between microbial signatures and host outcomes remain primarily associative. Establishing causality requires targeted interventions in well-controlled experimental systems.

### Microbiome-based therapeutics

Unlike the host genome, largely determined by parental inheritance, the human gut microbiome is constantly evolving in response to dietary, lifestyle, and pharmacological interventions (Rojo et al. 2017). This inherent plasticity positions the microbiome as a highly actionable target, which underpins the development of a diverse array of interventions broadly termed microbiome–based therapeutics. These therapeutics aim at restoring microbial equilibrium, selectively enriching beneficial taxa, or modulating specific community functions to improve host health. Current microbiome-based therapeutics encompass antibiotics, dietary modifications and prebiotics, traditional probiotics, next-generation live biotherapeutic products (LBPs), symbiotic microbial consortia, fecal microbiota transplantation (FMT), and postbiotics (Sorbara and Pamer 2022). Although the therapeutic potential of these approaches is well established, a consistent observation across modalities is the pronounced interindividual variability in treatment response.

#### Diet and prebiotics

Nutritional interventions represent the most fundamental means of shaping the gut microbiome to prevent and manage various health conditions, especially metabolic disorders (Wu et al. 2022). Diet directly influences substrate availability, thereby influencing microbial growth, gene expression, and metabolite production. Prebiotics, primarily non-digestible dietary fibers, selectively provide fermentable substances to a group of gut microbes and stimulate the production of short-chain fatty acids (SCFAs), which exert well-documented benefits on host metabolism and immune function (Mann et al. 2024). However, responses to identical dietary interventions differ substantially between individuals. In a recent randomized controlled trial of resistant-starch supplementation in patients with metabolic dysfunction-associated steatotic liver disease, baseline gut microbiome composition strongly stratified high responders from non-responders (Long et al. 2025). Only individuals harboring at baseline enriched fiber-degrading taxa and polysaccharide-utilization loci achieved a ≥ 30% reduction in intrahepatic triglyceride content and improvements in insulin sensitivity and fibrosis markers (Long et al. 2025). This marked variability arises from person-specific functional characteristics of the gut microbiome. Consequently, dietary and prebiotic interventions that demonstrate population-level benefits may produce attenuated or even negligible effects at the individual level.

#### Probiotics, LBPs, and symbiotic consortia

The utilization of live microorganisms as therapeutic agents represents a rapidly expanding frontier in microbiome–based therapeutics. Traditional probiotics typically include well-characterized species such as *Lactobacillus* spp., *Bifidobacterium* spp., or *Saccharomyces* spp. (Gareau et al. 2010), while LBPs consist of rationally selected or engineered strains intended to treat specific diseases (Pribyl et al. 2025, Kern et al. 2026). Prominent LBP candidates currently under investigation include *Akkermansia muciniphila* (Ioannou et al. 2025) and *Faecalibacterium prausnitzii* (Martín et al. 2023, Guarino-Vignon et al. 2026). A third modality, defined symbiotic consortia, consists of rationally assembled multi–strain communities that reconstitute key metabolic and protective functions through inter–species cross–feeding, competitive exclusion, and functional redundancy (Sorbara and Pamer 2022). These consortia offer greater compositional control and safety than undefined fecal preparations and demonstrate potential to remodel the gut ecosystem in preclinical studies (Furuichi et al. 2024, Pinheiro et al. 2025). These agents exert therapeutic effects through transient colonization, bioactive metabolite production, pathogen competition, and immunomodulation. Successful engraftment, however, remains highly individualized and dependent on the host baseline physiological and microbial characteristics (Zmora et al. 2018). Precision strategies to enhance colonization consistency are under active development, including human-milk-derived synbiotics, mucus-mimicking *in vitro* models, targeted delivery systems, and oxygen-tolerant strain engineering (Etienne-Mesmin et al. 2019, Button et al. 2023, Khan et al. 2023, Heavey et al. 2024).

#### FMT

Microbiome transplantation involves the transfer of a complete microbial ecosystem from a healthy donor to replace a dysbiotic community in a recipient. Ideally, this re-establishment restores beneficial microbial functions and reinstates essential crosstalk with the mucosal immune system. FMT is currently the most effective microbiome-based therapy for recurrent *Clostridioides difficile* infection (CDI) (Kassam et al. 2013). However, its efficacy in chronic inflammatory conditions, such as ulcerative colitis or metabolic syndrome, remains inconsistent (Paramsothy et al. 2017, Hanssen et al. 2021). Large-scale metagenomic analyses suggest that this heterogeneity arises from highly variable donor-strain engraftment and strain-level dynamics across diseases. Recipients with higher donor-strain engraftment are more likely to achieve clinical success, yet engraftment rates differ substantially depending on disease type, antibiotic preconditioning, and administration route (Ianiro et al. 2022). Complementary studies further reveal that recipient-specific factors, such as pre-FMT microbial richness, species abundances (e.g. *Bacteroides uniformis* and *Bacteroides vulgatus*), and donor–recipient complementarity, are the dominant drivers of strain coexistence, persistence, and colonization success (Schmidt et al. 2022). These person-specific functional traits must be characterized before transplantation to enable rational donor selection and preconditioning strategies, which moves FMT beyond a one–size–fits–all procedure toward a personalized microbial replacement therapy (Danne et al. 2021).

#### Postbiotics

Postbiotics are defined as non-viable microbial ingredients that confer beneficial physiological effects when administered through food and dietary supplements (Guglielmetti et al. 2025). Effective postbiotics contain microbial bioactive compounds, metabolic byproducts, cell components, and non-viable microorganisms. Unlike probiotics and LBPs, postbiotics offer a live-microbe-independent alternative that circumvents colonization requirements and provides superior batch-to-batch consistency. They exert beneficial effects primarily through direct interaction with host receptors and modulation of immune and metabolic pathways (Lavelle and Sokol 2020, la Cuesta-Zuluaga de et al. 2024, Zhao et al. 2024). The most studied postbiotics are SCFAs, notably butyrate, which triggers GLP-1 and PYY secretion *via* GPCR signalling to improve metabolic homeostasis (Hee van der and Wells 2021). Indole derivatives from tryptophan metabolism activate the aryl hydrocarbon receptor (AhR) to promote IL-22 production and mucosal barrier integrity (Agus et al. 2018). Oral supplementation with pasteurized *Akkermansia* muciniphila has shown safety and improvements in overweight and obese individuals with metabolic syndrome (Depommier et al. 2019). Similarly, heat-inactivated *Bifidobacterium bifidum* MIMBb75 substantially alleviates irritable bowel syndrome symptoms (Andresen et al. 2020). The broader field remains in its infancy. Prospective human trials are urgently needed to validate the therapeutic potential of postbiotics across diverse disease contexts.

### Factors influencing outcomes in microbiome-based personalized medicine

**Figure 2:**
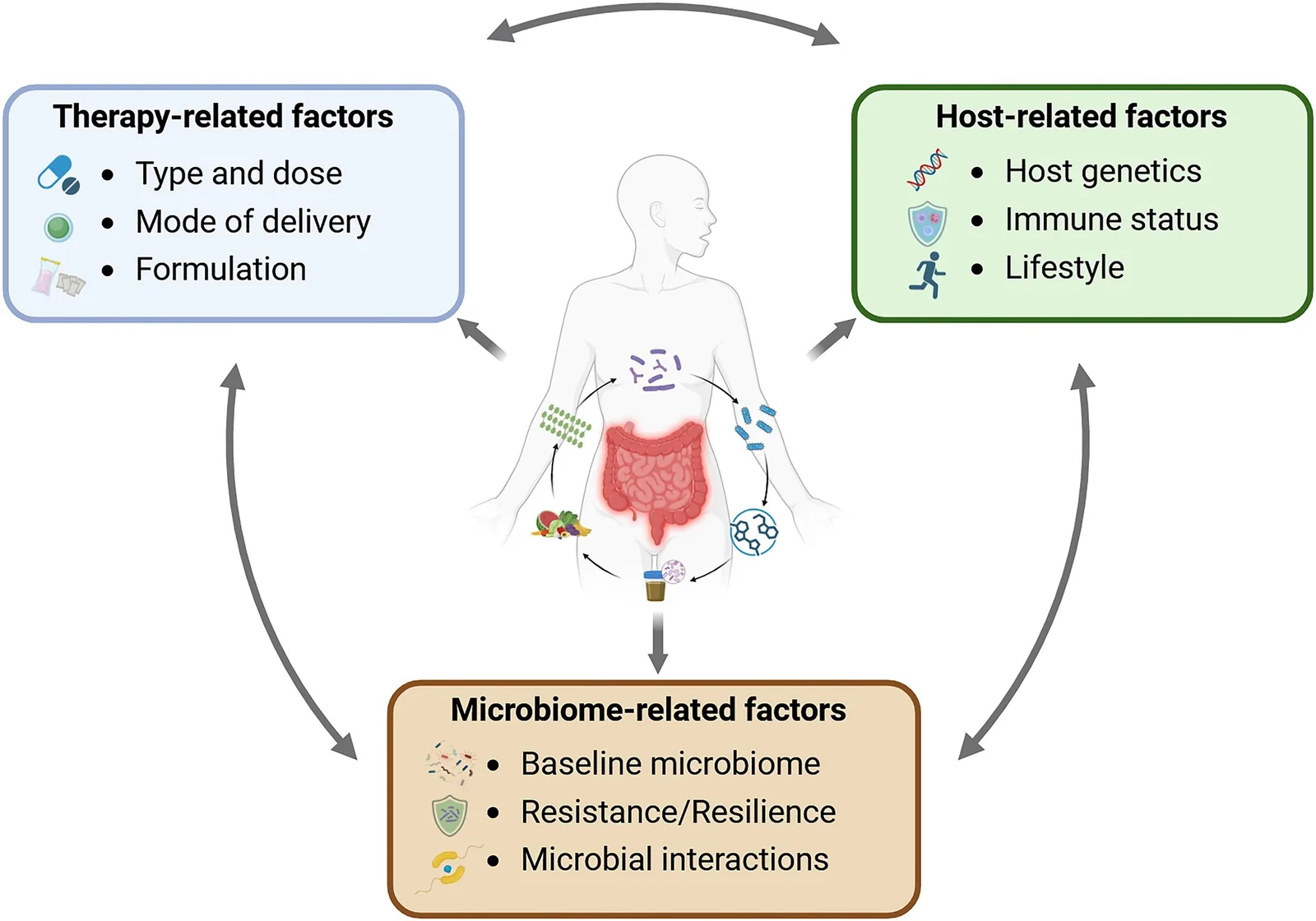
Determinants of inter-individual variability in microbiome-based personalized medicine. The efficacy of microbiome-based interventions is governed by a multi-dimensional landscape where the clinical outcomes emerge from the dynamic interplay of three primary axes. (1) Therapy-related factors: Clinical efficacy is dictated by molecular specificity (e.g. precision and broad-spectrum agents), dosage (dose-response relationships), and formulation (e.g. synergistic fiber blends). Targeted delivery mechanisms, such as engineered adhesion or encapsulation technologies, further modulate bioavailability and residence time. (2) Host-related factors: Variations in host genetics (e.g. *LCT, ABO*, and *FUT2* polymorphisms), immune status (e.g. baseline immune tone and barrier integrity), and lifestyle (e.g. exercise and habitual dietary patterns) serve as critical modifiers of the therapeutic response. (3) Microbiome-related factors: The pre-existing baseline composition acts as an ecological filter that determines the treatment efficacy. This is reinforced by intrinsic resistance and resilience mechanisms and community-scale microbial interactions, such as metabolic cross-feeding and the ratio of cooperative to competitive interactions. The convergence of these factors necessitates a transition from one-size-fits-all treatments toward integrated community-scale metabolic modeling for precision medicine.

The effectiveness of microbiome-based therapeutics is determined not only by the intervention itself but also by a highly complex and dynamic interplay among three principal elements: the therapeutic agent, the host, and the resident microbial ecosystem (Fig. 2). This multifactorial determinism underlies the divergent clinical, metabolic, and immunological outcomes observed in response to identical interventions. Systematically elucidating the key drivers of this variability is crucial to advancing beyond one-size-fits-all strategies toward truly personalized microbiome medicine.

#### Therapy-related factors

##### Type, dose, and time

The molecular specificity and dosage of a therapeutic agent are fundamental determinants of clinical efficacy and microbiome impact. Broad–spectrum antibiotics deplete both pathogenic and commensal bacteria, frequently leading to gut dysbiosis (Fishbein et al. 2023). In contrast, lolamicin, a precision Gram-negative-selective antibiotic that specifically inhibits the Lol lipoprotein transport system, retains potent activity against multidrug-resistant isolates while sparing anaerobic commensals (Muñoz et al. 2024). This targeted mechanism preserves microbiome diversity, maintains SCFA production, and prevents secondary CDI in murine models (Muñoz et al. 2024). A complementary class of macrocyclic peptide antibiotics further illustrates this principle by targeting the lipopolysaccharide transport machinery in *Acinetobacter*, thereby selectively killing Gram-negative pathogens while minimizing collateral damage to the gut ecosystem (Pahil et al. 2024). The importance of molecular specificity extends beyond antibiotics to prebiotics. Human studies reveal that specific prebiotic fibers elicit global, distinctive, and highly individualized alterations in gut microbial composition and molecular profiles (Lancaster et al. 2022). Critically, the chemical structure of a prebiotic, exemplified by the degree of polymerization of inulin, is a key determinant of its fermentation pattern and subsequent physiological impact (Li et al. 2020, Ariaee et al. 2024). Beyond molecular specificity, the magnitude of ecological perturbation follows a clear dose-response relationship. Higher cumulative concentrations and extended durations of exposure correlate with more profound shifts in the host metabolic landscape and a higher probability of transitioning the microbiome into a long-term dysbiotic state (Pennycook and Scanlan 2021, Fishbein et al. 2023). For FMT, timing relative to disease activity is clinically significant: administration during remission is associated with better engraftment and durability compared to active flares, and evidence supports multiple sequential infusions over single doses (Ianiro et al. 2022). For probiotics and LBPs, sustained daily dosing is generally required to maintain clinically relevant luminal concentrations, given the transient nature of colonization (Zmora et al. 2018). Kern et al. (2026) highlight temporal dosing precision as a key determinant of next-generation probiotic efficacy in personalized therapeutic contexts (Kern et al. 2026).

##### Mode of delivery

The route and method of delivery of a microbiome–based therapeutic influence its bioavailability, residence time, and pharmacodynamic profile. For probiotics and LBPs, achieving sufficient gut residence time remains a significant challenge, as most orally administered strains are rapidly cleared by peristalsis and host immune defenses. Heavey et al. (2024) engineered *S. boulardii* to display tuneable surface antibodies that bind to extracellular matrix proteins exposed during mucosal injury (Heavey et al. 2024). This targeted adhesion strategy extended its gut residence time compared to unmodified controls and achieved 100–fold increased probiotic concentrations within the colon in murine colitis models. Consequently, pharmacodynamic parameters, including barrier function, colonic cytokine expression profiles, and histological inflammation scores, were robustly improved and restored to healthy levels (Heavey et al. 2024). Beyond genetic engineering approaches, encapsulation technologies have emerged as vital delivery modalities. Single–cell encapsulation using biocompatible polysaccharides or lipid membranes protects probiotics from the harsh gastric environment and enables targeted release in the intestine (Zhao et al. 2024). Co–encapsulation strategies that combine probiotics with synergistic prebiotics or polyphenols enhance therapeutic effects by providing both live microbes and their required substrates (Ji et al. 2025). Bioinspired encapsulation platforms, which mimic natural protective structures such as bacterial cell membranes and biofilms, are now being developed to improve the survival, colonization, and targeted delivery of LBPs (Brittain and Finbloom 2025). Moreover, a new class of delivery systems, termed microbiome–active drug delivery systems (MADDS), leverages enzymatic and metabolic cues from the gut microbiota to achieve on–demand, site–specific release, adding a layer of precision that aligns therapeutic payload release with local microbial activity (Kamath, Ariaee et al. 2025). For FMT, the route of delivery (e.g. colonoscopy, upper endoscopy, nasointestinal tube, enema, and capsule) significantly affect donor strain engraftment and clinical outcomes. A meta–analysis of FMT trials found that higher donor strain engraftment was observed in individuals receiving FMT from multiple routes as well as in antibiotic-treated recipients with infectious diseases (Ianiro et al. 2022). Recent translational efforts have focused on overcoming the barriers to oral FMT capsule delivery through strategies such as lyophilization, enteric coating, and microencapsulation, which aim to preserve microbial viability and simplify large–scale application (Kamath et al. 2025).

##### Composition

The compositional structure of an intervention dictates both microbiome responses and downstream host physiology. In dietary interventions, the choice between whole foods and purified compounds produces distinct effects on the gut microbiome and immune system. For instance, a 17-week prospective study revealed that high-fiber diets primarily upregulate glycan-degrading enzymes with modest diversity shifts, whereas high-fermented-food diets robustly increase microbial diversity and suppress systemic inflammatory markers (Wastyk et al. 2021). Systematic blends of dietary fibers outperform mono-component formulations by supporting a broader array of beneficial taxa and maximizing SCFA production (Cantu-Jungles et al. 2025). The critical nature of composition is further highlighted in clinical contexts. In ketogenic models, the specific fiber content of ketogenic formulas, rather than the fat ratio or source, drives the metagenomic shifts required for seizure resistance (Özcan et al. 2025). For probiotics and LBPs, the compositional architecture is a primary determinant of therapeutic outcome. A striking demonstration comes from 123 very preterm infants, where two different probiotic products, Infloran^®^ and Labinic^®^, each drove the gut microbiome into distinct *Bifidobacterium*–enriched community types, with divergent stool metabolite profiles and functional implications for host–epithelium interaction (Beck et al. 2022). This divergence illustrates that even products with similar genus–level composition can produce profoundly different ecological and metabolic outcomes when their strain–level composition differs. Multi–strain consortia can broaden the functional repertoire, but the precise combination and ratio of strains can also introduce competitive exclusion or metabolic interference, making the specific composition of a consortium a critical efficacy determinant (Kern et al. 2026).

#### Host-related factors

##### Host genetics

Polymorphisms in genes encoding innate immune receptors, metabolic enzymes, and intestinal transporters modulate host responses to microbial metabolites and therapeutic interventions. Prolonged administration of the prebiotic inulin induced cholestatic liver injury and hepatocellular carcinoma in mice lacking Toll-like receptors 4 and 5 through unchecked microbial fermentation and accumulation of toxic secondary bile acids, whereas the same intervention improved metabolic parameters in wild-type animals (Singh et al. 2018). In a cohort of 5959 individuals, polymorphisms at loci such as *LCT, ABO*, and *FUT2* significantly modulated microbial taxa abundances and metabolic outputs, directly influencing incident cardiometabolic and inflammatory disease trajectories (Qin et al. 2022). Similarly, structural variations in the gut microbiome induced by host blood-group antigens and lactase persistence alter microbial gene content and metabolite production, creating individualized niches that determine responsiveness to prebiotics or probiotics (Zhang Q et al. 2024). In the context of FMT, host genetic factors govern donor-strain engraftment and long-term stability, with polymorphisms affecting mucosal immunity frequently associated with incomplete reconstitution or exaggerated inflammatory responses (Danne et al. 2021).

##### Immune status

The host immune status acts as a critical gatekeeper of engraftment efficiency, microbial stability, and therapeutic durability in microbiome-based interventions, particularly those involving live microorganisms or whole-community transplantation. In the context of FMT, immunodeficiency or severe inflammation frequently results in incomplete reconstitution, unstable engraftment, or clinical failure, whereas a balanced immune tone promotes tolerogenic dendritic cell maturation, regulatory T cell expansion, and sustained microbial integration (Littmann et al. 2021). Successful FMT recalibrates intestinal immune homeostasis by inducing the production of beneficial metabolites that drive anti-inflammatory cytokine profiles and reinforce epithelial barrier integrity (Yadegar et al. 2023). A recent single–cell immune atlas of FMT in CDI patients further demonstrates that successful treatment correlates with coordinated activation of regulatory, effector, and innate immune pathways, marked by transcriptional signatures of enhanced immune regulation and reduced cytotoxicity (Gao et al. 2026). This principle extends to probiotics and LBPs; while generally beneficial in immunocompetent hosts, administration in immunocompromised individuals requires caution due to risks of metabolic disturbances, excessive immune stimulation, or opportunistic infection (Kothari et al. 2019).

##### Lifestyle

Habitual lifestyle variables, including physical activity, tobacco use, alcohol consumption, and dietary patterns, profoundly modulate microbiome architecture and dictate susceptibility to therapeutic interventions. Regular moderate exercise promotes microbial diversity and enriches butyrate–producing taxa such as *F. prausnitzii* and *Roseburia*, thereby potentially improving the efficacy of probiotics and dietary interventions (Hawley et al. 2025). In contrast, cigarette smoking and chronic alcohol consumption induce dysbiosis characterized by a loss of commensal diversity and the expansion of pro-inflammatory species, thereby compromising barrier function and attenuating responsiveness to probiotics, prebiotics, and FMT (Bajaj 2019, Zhou et al. 2025). Furthermore, baseline dietary patterns represent a critical determinant of interventional outcomes. The magnitude of the microbial response to dietary fiber is often inversely proportional to habitual intake; individuals with low baseline fiber consumption exhibit more pronounced butyrogenic shifts and greater improvements in insulin sensitivity and inflammatory markers compared to high-fiber consumers (Holmes et al. 2022). This suggests a functional ceiling effect in microbiomes already adapted to fiber-rich substrates, whereas substrate-naive microbiomes retain greater plasticity for functional optimization.

#### Microbiome-related factors

##### Baseline microbiome composition

The baseline composition and functional capacity of the resident gut microbiome are primary determinants of therapeutic efficacy. This pre-existing ecological architecture acts as a selective filter, shaping susceptibility to perturbation and the likelihood of successful engraftment. Evidence from humanized gnotobiotic models further demonstrates that initial community structure critically influences microbial responses to antibiotic exposure: *Prevotella*/*Faecalibacterium*-dominated communities undergo marked destabilization following amoxicillin-clavulanate treatment, whereas *Bacteroides*/*Parabacteroides*-dominated communities remain significantly more stable (Lavelle et al. 2019). These findings have been extended to humans, where specific pre-treatment taxa predict the extent and duration of antibiotic-induced disruption (Rashidi et al. 2021). Similarly, baseline composition governs mucosal colonization resistance during probiotic supplementation. Engraftment is highly individualized, with endogenous microbes frequently limiting the establishment of exogenous strains (Zmora et al. 2018). In FMT, the recipient’s native bacterial composition critically influences clinical success; for instance, baseline *Alistipes* abundance negatively predicts prognosis in inflammatory bowel disease, while high baseline *Prevotella* abundance correlates with diminished metabolic improvement in obesity (Zhang et al. 2024, Zhao et al. 2025).

##### Microbial resistance and resilience

The resistance (the ability of a microbial community to resist perturbation) and resilience (the capacity to recover after perturbation) of the gut microbiome are key drivers of long-term therapeutic success or failure. Enrichment of gatekeeper species such as *B. uniformis* and *B. vulgatus* in the recipient microbiota can inhibit donor-strain colonization through competitive niche occupation, bacteriocin production, and bile-acid conjugation, frequently resulting in clinical non-response of FMT (Ianiro et al. 2022, Schmidt et al. 2022). This resistance is further reinforced by lantibiotic-producing bacteria, which generate narrow-spectrum antimicrobials to limit the establishment of exogenous strains (Cole et al. 2025). Following antibiotic or dietary challenge, the microbiome exhibits individualized recovery trajectories dictated by pre-existing community architecture (Fassarella et al. 2021). Chemotherapeutic agents can impose an analogous selective pressure, and baseline microbial composition has been shown to govern the resilience of the gut ecosystem during cancer treatment, influencing both gastrointestinal toxicity and treatment efficacy (Chan et al. 2026). While certain taxa display intrinsic resistance mediated by efflux pumps or metabolic bypass pathways, others undergo transient collapse followed by resilient regrowth supported by cross-feeding networks (de la Cuesta-Zuluaga et al. 2024). Global metagenomic analyses confirm that these resistance and resilience states are defined by the interplay of host factors and environmental exposures, resulting in stable microbiome configurations that predict differential responses to dietary and microbial therapies (Tap et al. 2023).

##### Microbial interactions

The gut microbiome functions as a densely interconnected network where metabolic cross-feeding and resource competition dictate community stability and therapeutic efficacy. Indole-3-lactic acid produced by *Lactobacillus* spp. serves as a precursor for secondary degraders which convert it into AhR ligands, such as indole-3-propionic acid and indole-3-acetic acid; disruption of this network impairs mucosal barrier function in murine colitis models (Wang et al. 2024). Dietary fiber directs similar tryptophan metabolism through defined cross-feeding cascades, while dietary phytate metabolism in the gut is entirely dependent on sequential microbial cross-feeding between primary and secondary degraders (Sinha et al. 2024, Vos et al. 2024). These mechanistic insights have enabled the rational design of synthetic consortia, such as *Bacteroides xylanisolvens* and *Clostridium butyricum*, which exploit mutualistic cross-feeding to enhance butyrate production and improve clinical metabolic outcomes (Qiao et al. 2025). Interestingly, consumer-resource modeling of the human gut microbiome reveals that healthy communities are dominated by competitive interactions, whereas dysbiosis is characterized by a shift toward tightly connected cooperative consortia (Corral López et al. 2026). The Ecological Network Balance Index (ENBI), which quantifies the ratio of cooperative to competitive interactions, reliably distinguishes health from disease states (e.g. IBD, CDI, and colorectal cancer); higher ENBI values correlate with disease progression and reduced therapeutic responsiveness (Corral López et al. 2026). Collectively, these dynamics necessitate the integration of community-scale metabolic modeling into pre-treatment assessments to enable truly personalized microbiome interventions.

### Advancing microbiome-based personalized medicine through *in vitro* platform

Achieving a truly personalized approach requires mechanistic dissection of the complex interactions that shape the treatment responses at the individual level. Conducting such studies *in vivo*, encompassing human clinical trials and animal models, remains indispensable for capturing holistic microbiome-host dynamics, systemic physiological effects, and real-world therapeutic outcomes. However, they are inherently limited by high costs, prolonged timelines, ethical constraints, and confounding variables. Animal models face additional challenges of species differences, husbandry practices, gnotobiotic facilities, and experimental protocols (Moossavi et al. 2022). Even human microbiota-associated models introduce ecological mismatches and physiological differences in immune and metabolic systems (Arrieta et al. 2016). There is also growing societal and regulatory pressure to reduce or replace animal testing.

**Figure 3:**
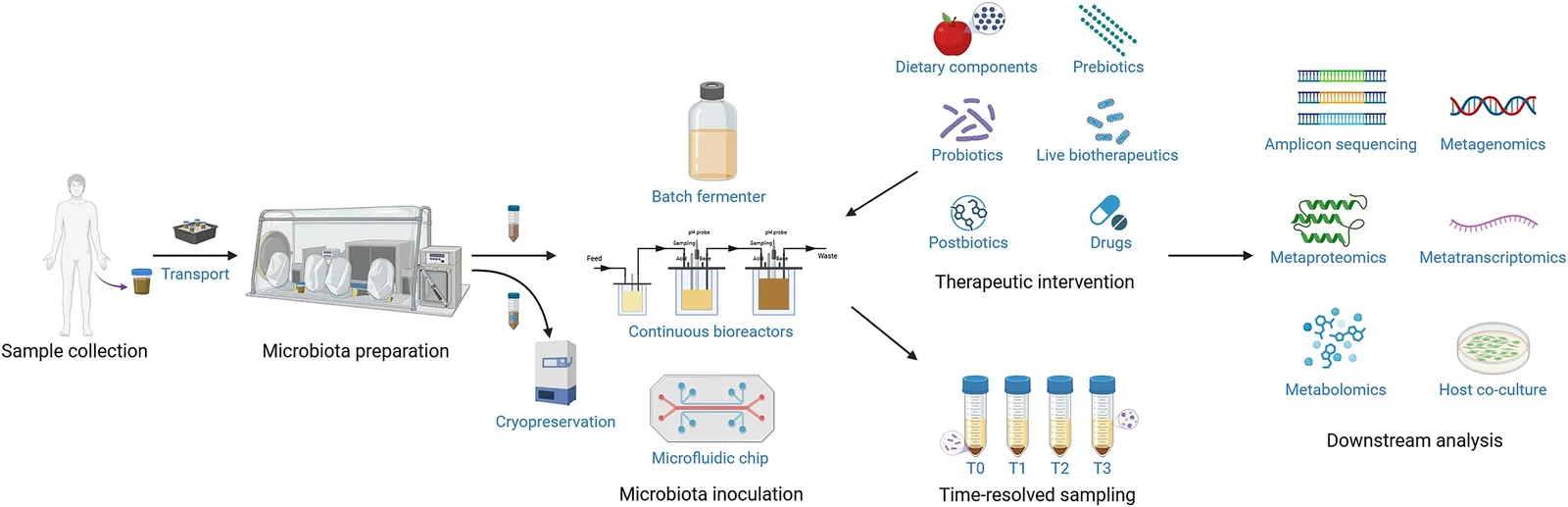
Standardized experimental workflow for *in vitro* gut microbiome studies. The pipeline integrates six critical stages. (1) Sample collection: Fecal samples serve as the primary and most practical inoculum source. Due to the inherent instability of fresh fecal material, specimens are collected using anaerobic transport systems and oxygen-scavenging sachets. To minimize metabolic drift and suppress enzymatic activity, samples are maintained at 4°C or on ice. (2) Microbiota preparation: To prevent the loss of oxygen-sensitive taxa, all sample manipulations are conducted within specialized anaerobic workstations. To mitigate the impact of topographical heterogeneity within fecal matter and reduce reducing intra-sample variability, thorough homogenization of the entire specimen is performed prior to subsampling. While fresh cultures are preferred for immediate applications, long-term biobanking in deoxygenated glycerol buffer at –80°C ensures longitudinal experimental reproducibility and logistical efficiency. (3) Microbiota inoculation: The selection of an appropriate *in vitro* platform is dictated by the specific research objective. Operational parameters, including anaerobic atmosphere, pH gradients, culture media composition, and nutrient flux, are precisely calibrated to recapitulate the physiological features of the target population. (4) Therapeutic intervention: Systematic application of microbiome-modulating agents (e.g. dietary components, prebiotics, probiotics, live biotherapeutic products, postbiotics, and drugs). Optimized experimental designs and stratified replicate organization are employed to enhance statistical power and facilitate robust outcome interpretation. (5) Time-resolved sampling: High-resolution longitudinal sampling is implemented to characterize the successional dynamics of the microbial community and the associated metabolic transitions over time. (6) Downstream analysis: Downstream characterization utilizes a suite of high-throughput assays, including amplicon sequencing, shotgun metagenomics, metaproteomics, metatranscriptomics, metabolomics, and host co-culture systems to dissect ecological and functional signatures. These multi-omic datasets are integrated using advanced computational, bioinformatic, and artificial intelligence frameworks to identify candidate biomarkers and therapeutic targets.

In this context, *in vitro* gut models have emerged as essential platforms. A typical *in vitro* workflow follows a standardized and reproducible sequence (Fig. 3): (1) collection of fresh human fecal or mucosal samples from individual donors or patients; (2) preparation and cryopreservation of microbiota inocula under specialized anaerobic workstations; (3) inoculation of the microbiota into controlled culture systems (e.g. batch fermenters, continuous bioreactors, and gut-on-a-chip platforms) under physiologically relevant conditions (e.g. anaerobic environment, controlled pH, temperature, and nutrient flow); (4) application of the therapeutic intervention (e.g. dietary components, prebiotics, probiotics, LBPs, postbiotics, and drugs) at clinically relevant concentrations; (5) time-resolved sampling over hours to days to capture dynamic community shifts; and (6) comprehensive biological readouts *via* multi-omics approaches (e.g. 16S rRNA, shotgun metagenomics, metaproteomics, metatranscriptomics, metabolomics, and host co-culture assays). By enabling the systematic evaluation of person-specific responses, *in vitro* approaches bridge critical knowledge gaps and accelerate the translation of microbiome science into personalized medicine.

#### Integrated biomimetic systems facilitating human-relevant data generation

*In vitro* models excel at isolating host-independent microbial dynamics and biochemical mechanisms that are often obscured *in vivo*, offering critical insights into therapy–microbiome interactions under controlled conditions. By directly inoculating these systems with microbiota derived from individual human donors, researchers can faithfully reconstitute person-specific microbial communities and their functional potential while eliminating confounding host influences, such as epithelial absorption, immune signalling, and systemic metabolism (Li et al. 2019, Huang et al. 2025). This donor-matched strategy enables precise investigation of inter-individual variability in microbial responses to therapeutic agents, which reveals how baseline community structure determines metabolite production, cross-feeding networks, and overall functional output.

Microbe–host interaction platforms that incorporate human intestinal epithelial cells, organoids, or gut-on-a-chip devices recreate physiologically relevant microenvironments, including oxygen gradients, mucus layers, and mechanical shear stress (Zhang et al. 2021, Moossavi et al. 2022). These advanced co-culture systems allow direct interrogation of microbiome–host crosstalk at the molecular level, such as changes in epithelial barrier integrity and immune activation pathways triggered by microbial stimuli (Özkan et al. 2024, Harter et al. 2025). A particularly powerful extension of this approach is to use patient-derived samples, cells, or tissues, although access to such material can be limited. Patient-specific intestinal organoids or primary epithelial monolayers generated from endoscopic biopsies or surgical resections enable truly individualized modeling, allowing researchers to investigate how a given patient’s own microbiome interacts with their own genetically and disease-specific epithelial and immune cells (Moossavi et al. 2022, Özkan et al. 2024). This patient-matched platform provides mechanistic insights into personalized disease processes and reveals how specific microbial communities or therapeutic agents differentially affect that individual’s epithelium at the transcriptional, proteomic, and functional levels. By linking donor-specific microbial communities with patient-derived host tissues, these systems generate human-relevant mechanistic data for the rational design of truly personalized microbiome-based therapies.

#### High-throughput screening approaches facilitating large-scale evaluation

*In vitro* models provide superior scalability for systematic testing of multiple interventions in a time- and cost-effective manner. By leveraging miniaturization and automation, these systems enable the parallel screening of extensive libraries, including drugs, prebiotics, probiotics, or dietary components, under highly standardized conditions. This approach addresses the ethical and financial constraints that often limit the feasibility of large-scale *in vivo* studies. Consequently, researchers can simultaneously evaluate complex intervention libraries against individual or cohort-specific microbial communities, significantly accelerating the identification and optimization of microbiome-modulating agents (Maier et al. 2018, Baert et al. 2021). When integrated with multi-omics workflows (metagenomics, metaproteomics, metatranscriptomics, and metabolomics), these platforms generate high-resolution datasets that link community-level taxonomic shifts to specific gene expression changes and metabolite outputs in real time (Li et al. 2020, Ricaurte et al. 2024). This integrated approach clarifies the biochemical mechanisms driving host-specific responses.

*In vitro* models facilitate direct comparative analysis of microbiota obtained from different individuals, populations, or disease states under identical experimental conditions. By inoculating standardized culture systems with fecal samples from healthy donors versus patients, or from therapeutic responders versus non-responders to a given therapy, researchers can rapidly identify community-specific susceptibilities, resilience patterns, and predictive baseline features that determine therapeutic outcome (Vigsnaes et al. 2013, Javdan et al. 2020). These scalable platforms serve as essential tools for bridging the gap between individualized variability and population-level discovery, while reducing reliance on animal testing.

#### Precise biomarker discovery driving new therapeutics

The controlled nature of *in vitro* models provides an ideal environment for precise analysis of microbiome signatures and the discovery of indicative biomarkers that can guide personalized interventions. Unlike *in vivo* settings, where many metabolites are rapidly absorbed or metabolized by the host, *in vitro* models allow direct measurement of microbial metabolic products, uncovering otherwise elusive microbiome–metabolome links that are critical for understanding therapeutic mechanisms (Treuren Van and Dodd 2020).

A critical application of this precision is the analysis of intestinal gases, which are challenging to quantify accurately *in vivo* due to rapid diffusion and host consumption. *In vitro* fermentation systems facilitate the detection and quantification of gas composition (e.g. hydrogen, methane, carbon dioxide, and hydrogen sulfide), which provides direct readouts of fermentation efficiency, cross-feeding dynamics, community metabolic states, and host health (Ou et al. 2015, Larik et al. 2024). For instance, hydrogen serves as both a non-invasive metric of microbial activity and a metabolic regulator that shapes community architecture by supporting specific lithotrophic taxa (Smith et al. 2019). Disruption of hydrogen cycling has been linked to gastrointestinal disorders, infections, and even cancer, underscoring its potential as a clinically relevant biomarker (Welsh et al. 2025). Hydrogen also serves as a substrate for microbial transformations that generate bioactive molecules; gut bacteria such as *Gordonibacter pamelaeae* and *Eggerthella lenta* convert glucocorticoids into progestins in the presence of hydrogen, revealing previously unrecognized microbiome-mediated steroid metabolism pathways with potential endocrine and inflammatory implications (McCurry et al. 2024).

The mechanistic precision of *in vitro* models further enables the discovery of complex interaction networks that would be intractable *in vivo*. By controlling microbial community composition and nutrient availability, researchers can dissect causal relationships between specific microbial activities and host outcomes, moving beyond the correlative associations that dominate human cohort studies. In particular, *in vitro* platforms have demonstrated how dietary fiber directs microbial tryptophan metabolism through defined cross-feeding cascades (Sinha et al. 2024) and how nutrient and resource competition predicts microbiome restructuring under drug perturbations and the assembly of gut bacterial communities (Ho et al. 2024, Shi et al. 2025). These controlled studies establish causal links between microbial metabolic networks, resource competition, and functional outputs, allowing the identification of robust biomarkers that can be translated into clinical diagnostics and personalized therapeutic strategies.

### *In vitro* gut models for microbiome-based personalized medicine

**Figure 4:**
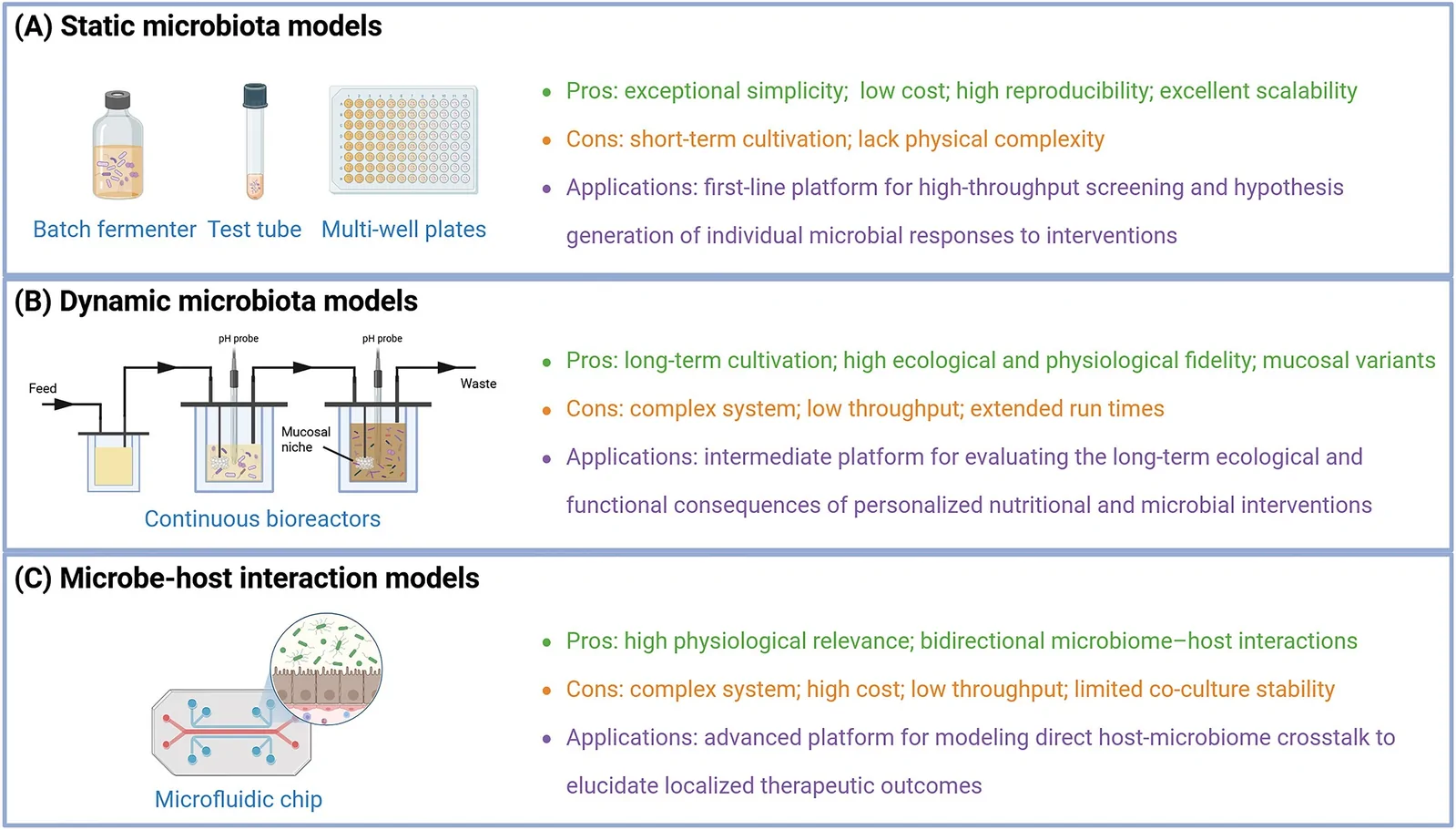
Overview of *in vitro* gut models and their applications in microbiome-based personalized medicine. (A) Static microbiota models: Consisting of closed batch fermenters, test tubes, or multi-well plates operated under strictly anaerobic conditions for short durations (24–48 h), these systems are highly reproducible, cost-effective, and easily scalable. They serve as an indispensable tool for high-throughput screening, enabling the rapid evaluation of personalized microbial responses to large panels of dietary compounds, prebiotics, and drugs, as well as the prediction of individual metabolic outputs when coupled with computational approaches. (B) Dynamic microbiota models: These platforms (e.g. SHIME^®^, TIM, PolyFermS, and MBRA) overcome the limitations of static systems by incorporating continuous nutrient flow, waste removal, pH gradients, controlled transit times, and simulated mucosal compartments. Enabling long-term cultivation (days to weeks) with high ecological fidelity, they act as an intermediate platform to evaluate the long-term ecological succession, spatial colonization, and individualized functional consequences of nutritional and microbial interventions. (C) Microbe-host interaction models: Representing the most advanced systems (e.g. HuMiX and Gut-on-a-chip), these platforms utilize microfluidic architectures and controlled oxygen gradients to reconcile the fundamentally opposing environmental needs of living mammalian cells and strict anaerobic bacteria. By simulating mechanical forces like peristalsis and fluid shear stress, they enable direct live host-microbe co-culture, which provides a powerful platform for elucidating host-microbiome crosstalk and localized therapeutic outcomes.

The transition from descriptive microbiome associations to targeted personalized interventions requires experimental systems that can capture the functional complexity of the human gut ecosystem while maintaining experimental control. Advances in human gut microbiota culturing have driven the development of three distinct paradigms of *in vitro* culturing systems: static microbiota models, dynamic microbiota models, and microbe-host interaction models (Fig. 4). These systems are not mutually exclusive but rather form a progressive toolkit that enables researchers to address different types of research questions, ranging from high-throughput screening of microbiome-modulating agents to long-term simulation of microbial adaptation under gut-like conditions and mechanistic dissection of bidirectional host-microbiome crosstalk. Together, they provide a comprehensive platform for dissecting person-specific microbiome responses to therapeutic interventions, identifying predictive biomarkers, and accelerating the translation of microbiome science into personalized clinical applications.

#### Static microbiota models

Static microbiota models represent the most fundamental and widely accessible *in vitro* systems for studying human gut microbiome. They consist of closed batch reactors, test tubes, or multi-well plates operated under strictly anaerobic conditions for short durations (typically 24–48 h). Their core advantages lie in exceptional simplicity, low cost, high reproducibility, and compatibility with automation and miniaturization, making them uniquely suited for high-throughput screening of microbial responses to interventions such as drugs, probiotics, prebiotics, and dietary compounds (Fig. 4A).

Stool-derived inocula are the most used starting material due to their non-invasive collection, high convenience, and abundant biomass. When cultured in 96-well plates, stool-derived microbial communities remain phylogenetically complex and highly reproducible, retaining inter-individual taxonomic and functional variation while recapitulating *in vivo*-like responses to perturbations such as antibiotics (Aranda-Díaz et al. 2022). Similarly, the scalable MiPro 96-deep-well plate model maintains both compositional and functional profiles of individual gut microbiomes (Li et al. 2019). For dietary intervention studies, standardized batch fermentation protocols using human fecal inocula offer a scalable and reproducible means to evaluate how specific food substrates modulate microbiota composition and function (Pérez-Burillo et al. 2021). When coupled with community-scale metabolic modeling, these platforms can predict personalized SCFA production profiles, bridging dietary input with host-metabolite availability (Quinn-Bohmann et al. 2024). In pharmacomicrobiomics, platforms such as the Microbiome-Derived Metabolism (MDM)-Screen map strain-level drug biotransformation using personalized gut microbial communities in static culture, directly linking individual microbiome composition to differential drug metabolism (Javdan et al. 2020). Complementing this, the RapidAIM framework integrates *in vitro* culturing with metaproteomics for high-throughput profiling of functional responses to xenobiotics at the individual microbiome level (Li et al. 2020). Beyond xenobiotics, stool-derived models serve as critical tools for deciphering ecological assembly rules, enabling data-driven predictions of colonization outcomes for introduced microbes or LBPs (Wu et al. 2024).

While stool-derived models offer high physiological relevance, their undefined complexity can obscure precise causal relationships. To overcome this, researchers employ defined synthetic consortia for highly controlled mechanistic studies (Leeuwen van et al. 2023). These reduced-complexity models enable precise dissection of microbial dynamics, interspecies cross-feeding, and gene–function relationships. A landmark application of this approach was the systematic screening of over 1000 non-antibiotic drugs against a panel of individual bacterial isolates, which revealed that over 24% of common medications, including cardiometabolic and psychiatric drugs, exert unintended antimicrobial effects (Maier et al. 2018). To ensure rigorous experimental reproducibility, a standardized reference model, the Simplified Human Intestinal Microbiota (SIHUMI), has been established. When combined with advanced tracking methods such as targeted qPCR, this platform enables strain-level quantification and serves as a versatile tool for dissecting the functional dynamics of the gut microbiome in response to therapeutic and environmental perturbations (Ríos Colombo et al. 2026).

Although static models cannot replicate continuous flow, pH gradients, or mucus-associated niches of the human colon, their scalability, mechanistic precision, and ability to maintain donor-specific functional activities make them an indispensable first-line tool for hypothesis generation, large-scale screening, and the identification of candidate interventions that warrant testing in more physiologically complex dynamic or co-culture systems. When combined with multi-omics readouts and computational modeling, static platforms provide a robust entry point into personalized microbiome research.

#### Dynamic microbiota models

Dynamic microbiota models address many physiological limitations of static systems by incorporating continuous nutrient inflow, waste removal, pH gradients, peristaltic mixing, and controlled transit times. These features enable long–term cultivation (days to weeks) and realistic simulation of colonic transit, mucus–associated niches, and regional differences along the gut, providing higher ecological fidelity than static systems (Fig. 4B). Prominent dynamic models include the Simulator of the Human Intestinal Microbial Ecosystem (SHIME^®^) and its mucosal variants (M-SHIME^®^), the TNO Gastrointestinal Models (TIM–1 and TIM–2), the PolyFermS platform, and the Multi–Bioreactor Array (MBRA) and its mucosal variants (MBRA 3.0).

Dynamic platforms are uniquely suited for investigating temporal ecological processes, including microbial adaptation to repeated dietary or pharmacological challenges, probiotic/prebiotic-driven community succession, and engraftment dynamics after FMT. Utilizing the PolyFermS platform, researchers have demonstrated that dietary fibers exert distinct prebiotic effects that are significantly influenced by baseline microbiota composition (Poeker et al. 2018). Furthermore, *in vitro* dynamic modeling has revealed that the physiological impact of dietary fiber on colitis susceptibility is highly individualized; the same fiber intervention can either protect against or exacerbate inflammation depending on the recipient’s pre–existing microbial community structure (Bonazzi et al. 2024). An MBRA–based *in vitro* microbiota model has been established that successfully recapitulates and predicts individualized sensitivity to dietary emulsifiers, identifying specific baseline microbial signatures susceptible to emulsifier-induced dysbiosis (Rytter et al. 2025). Beyond substrate availability, dynamic systems highlight the critical role of gut mechanics. Manipulating simulated transit times *in vitro* has revealed that microbial growth rates, nutrient use efficiency, and adaptive responses are highly sensitive to *in vivo* transit dynamics, underscoring the necessity of factoring inter-individual physiological differences into personalized nutritional interventions (Minnebo et al. 2023).

A pivotal advancement in dynamic models is the integration of simulated mucus layers, such as the mucin-agar compartments utilized in M-SHIME^®^ or MBRA 3.0 (Wiele Van de et al. 2015, Duquesnoy and Chassaing 2026), to study mucosal colonization. These mucosal models have revealed profound disease-associated functional alterations. For instance, the microbiota of ulcerative colitis patients in relapse and remission exhibits defective mucosal colonization by *Lactobacillus* and *Bifidobacterium* species alongside altered metabolic outputs, suggesting that spatial colonization defects fundamentally drive disease pathophysiology (Vigsnaes et al. 2013). The probiotic yeast *Saccharomyces cerevisiae* CNCM I-3856 has been shown to exert multi-targeted inhibitory activity against enterotoxigenic *Escherichia coli* pathogenesis across TIM-1 and M-SHIME^®^ models, maintaining its efficacy regardless of inter-individual microbiome variability (Roussel et al. 2021). In a M-SHIME^®^ model, arabinoxylans, inulin, and *Lactobacillus reuteri* have been shown to repress the growth of adherent-invasive *E. coli* within the simulated mucus compartment (Abbeele Van den et al. 2016). For Crohn’s disease, supplementing continuous fermentation models with targeted butyrate-producing consortia effectively enhances butyrate availability and colonization capacity in the mucus environment, providing a mechanistic rationale for microbiome-based adjunct therapies aimed at restoring epithelial barrier integrity (Geirnaert et al. 2017).

Beyond adult dysbiosis, dynamic models have also been applied to study early–life microbiome interventions. In a recent breakthrough using a dynamic colonic model, the human milk oligosaccharide 2′-fucosyllactose combined with cross-feeding bifidobacteria successfully reshaped the dysbiotic gut microbiota of infants with atopic dermatitis (Qi et al. 2025). When this *in vitro*-reshaped microbiota was transplanted into an oxazolone-induced murine model, it effectively prevented atopic dermatitis symptoms via the activation of host retinol metabolism, illustrating the translational pipeline from *in vitro* pre-conditioning to *in vivo* efficacy (Qi et al. 2025). To balance physiological complexity with experimental reproducibility, dynamic models are evolving to incorporate defined minimal microbiomes. By employing rationally designed consortia that exhibit core dynamic metabolic interactions, researchers can precisely map trophic networks and cross-feeding relationships without the confounding noise of undefined fecal matter (Shetty et al. 2022). Concurrently, the expansion of reproducible small intestinal microbiota models integrated into the SHIME^®^ system now enables the region-specific investigation of microbial dynamics along the entire length of the gastrointestinal tract (Deyaert et al. 2023).

Compared with static models, dynamic systems are inherently more complex, requiring specialized equipment, trained operators, and extended run times, which limits their throughput and the number of tested conditions or samples that can be processed in parallel. Nevertheless, their ability to maintain stable, metabolically active communities under physiologically relevant conditions makes them an indispensable intermediate platform for evaluating the long-term functional consequences of interventions. Dynamic systems bridge the gap between high–throughput static screening and costly *in vivo* studies, providing a framework for optimizing microbiome–based personalized therapies prior to clinical translation.

#### Microbe-host interaction models

Microbe-host interaction models represent the most physiologically integrative class of *in vitro* systems for microbiome research. Unlike static or dynamic models designed primarily to study microbial community behavior, co-culture platforms incorporate living human-derived cells (e.g. intestinal epithelial cells, immune cells, organoids, and control- and patient-derived tissues) to enable direct investigation of microbiome–host interactions (Fig. 4C). These models are uniquely positioned to address questions that purely microbial systems cannot answer, such as how gut microbes influence epithelial barrier integrity, immune activation, mucus production, and inflammatory signaling, and how host factors in turn shape microbial community behavior and therapeutic outcomes.

A primary technical challenge in developing these models is reconciling the fundamentally opposing environmental requirements of mammalian cells and gut microbes. Mammalian cells require oxygen and carbon dioxide for survival and proliferation, whereas most colonic commensals are strict anaerobes that are highly sensitive to even trace levels of oxygen. Recent technological innovations have largely overcome this barrier through dual-chamber designs that physically separate the microbial and host compartments, microfluidic oxygen control that establishes physiological oxygen gradients across the epithelial barrier, and flow-based architectures that maintain anoxic conditions in the luminal compartment while perfusing oxygenated medium to the basolateral side (Sardelli et al. 2021, Li et al. 2022).

Leveraging these microfluidic solutions, pioneering platforms such as the HuMiX (Human-Microbial Crosstalk) device were among the first to successfully co-culture human intestinal epithelial cells with strict anaerobic gut bacteria at a simulated gastrointestinal interface. This platform faithfully recapitulates *in vivo*-like transcriptional, metabolic, and immunological responses in human intestinal epithelial cells during anaerobic co-culture with commensals like *Lactobacillus rhamnosus* GG and *Bacteroides caccae* (Shah et al. 2016). Building upon this framework, researchers utilized HuMiX to demonstrate that the co-culture of *Fusobacterium nucleatum* with patient-derived colorectal cancer cells promotes tumour progression (Ternes et al. 2022). Specifically, this bacterium induces a metabolic shift toward enhanced glutamine metabolism and increased secretion of the microbial metabolite formate, which acts as an oncometabolite to drive cancer invasion via AhR signalling and increased cancer stemness (Ternes et al. 2022). Furthermore, integrated *in vitro* and *in silico* modeling has been successfully applied to such systems to delineate the molecular effects of targeted synbiotic regimens on colorectal-cancer-derived cells (Greenhalgh et al. 2019).

Gut-on-a-chip and intestine-on-a-chip platforms represent the most advanced evolution of microbe-host interaction models. These devices integrate primary human intestinal cells with tissue engineering and mechanical simulations of peristalsis and fluid shear stress to recapitulate essential structural and functional features of the human intestine, including a polarized epithelium, a protective mucus layer, dynamic fluid flow, and controlled oxygen gradients (Özkan et al. 2024). Crucially, simulating this mechanical deformation has proven necessary for accurately modeling intestinal bacterial overgrowth and subsequent inflammatory cascades (Kim et al. 2016). A landmark study developed an anaerobic intestine-on-a-chip capable of sustaining a complex human gut microbiome in direct contact with living intestinal epithelium for extended periods, maintaining both epithelial barrier function and microbial community stability (Jalili-Firoozinezhad et al. 2019). Moving beyond basic epithelial interfaces, immune-competent gut micro-physiological systems now permit the long-term co-culture of next-generation probiotics like *F. prausnitzii*, revealing their capacity to directly modulate inflammation-related gene expression in the human colonic epithelium (Zhang et al. 2024).

The translational applications of these devices are expanding rapidly into precision medicine and high-throughput screening. For instance, a sophisticated gut-on-a-chip incorporating human fecal samples and peristalsis-like movements successfully predicted individual patient responses to immune checkpoint inhibitors in melanoma by identifying specific gut inflammation-related biomarkers and microbial signatures that distinguish responders from non-responders (Ballerini et al. 2025). Additional innovations include 3D gradient hydrogel systems for investigating microbiome–drug interactions to guide therapeutic decisions (Zheng et al. 2023), as well as intelligent intestine-on-a-chip platforms that leverage machine learning for the rapid screening of probiotics with targeted enteritis-relieving functions (Wu et al. 2024).

By integrating complex microbiota with living host cells in a highly controlled setting, microbe-host interaction models bridge the critical gap between microbial community dynamics and host physiology. They offer a powerful platform for studying personalized immune modulation, barrier integrity, metabolite signaling, and disease mechanisms in a human-relevant context. As these technologies continue to mature, they are expected to play an increasingly central role in translating microbiome signatures into clinically actionable interventions tailored to individual patients.

## Challenges and outlook

Although *in vitro* gut models have emerged as powerful tools for advancing microbiome-based personalized medicine, significant technical, biological, and conceptual limitations still restrict their translational impact. Closing the gap between controlled experimental platforms and the dynamic complexity of the human gut will require continued innovation in model design, biological integration, analytical precision, and computational coupling. Here, we delineate key challenges and propose strategic directions to enhance physiological fidelity and clinical relevance.

### How can *in vitro* models better preserve the *ex vivo* human gut microbiota?

A fundamental challenge in *in vitro* gut modeling is faithfully preserving the taxonomic composition and functional activity of the original microbiome during cultivation (Vrancken et al. 2019). Even under optimized anaerobic conditions, inoculated communities often undergo substantial compositional shifts with selective enrichment of fast-growing generalists and progressive loss of functionally important taxa. Such compositional shifts can profoundly alter community metabolism and ecological behavior, thereby compromising the biological relevance and interpretability of experimental results.

To address these limitations, several complementary strategies are being developed:

- **Optimizing pre-analytical workflows:** The trajectory of an *in vitro* community is heavily influenced by events that occur before the inoculum reaches the culture vessel (Gratton et al. 2016). Rigorous standardization of fecal sample collection, transport, processing, and cryopreservation protocols is essential. Maintaining an uninterrupted anaerobic chain from the point of defecation to inoculation minimizes oxygen-induced community drift. The choice of cryopreservant also matters (Biclot et al. 2022), which differentially affect the recovery of specific taxa, and optimization for the target community is warranted. Importantly, inter-individual variability in the rate of community decay post-collection suggests that personalized pre-analytical protocols may eventually be required to standardize *in vitro* outcomes. Machine learning models trained on paired fresh versus processed samples could guide this personalization.
- **Advancing physicochemical control:** Replicating the physicochemical landscape of the human colon requires more than bulk anaerobiosis. The gut features steep oxygen gradients, with near-anoxic conditions in the lumen and microaerobic zones at the mucosal surface. Sophisticated anaerobic workstations, microfluidic oxygen-gradient chambers, and real-time dissolved oxygen monitoring are necessary to sustain obligate anaerobes while providing physiologically relevant oxygen tensions for microaerophiles. Additionally, personalizing key operational parameters, such as pH, nutrient inflow gradients, transit time, and metabolite removal rates, according to donor-specific physiology can help maintain individualized microbiota signatures throughout the culture period. For instance, individuals differ in colonic transit time, a parameter that shapes community composition and metabolic output (Minnebo et al. 2023); dynamic bioreactors that simulate donor-specific transit kinetics would therefore preserve greater interpersonal variability than one-size-fits-all flow rates.
- **Designing culture media rationally:** Culture media should closely mimic the biochemical environment of the human gut by incorporating host-derived factors (e.g. mucins and bile acids), complex dietary substrates (e.g. resistant starch and pectin), and essential cofactors. Mucins serve dual roles as nutrients for specialized degraders and as attachment surfaces that promote biofilm-like community structures, yet they are often omitted from standard media. Bile acid pools vary markedly between individuals, and tailoring the bile acid composition of the medium to donor profiles could preserve the selective pressure that maintains bile acid-metabolizing taxa (Li et al. 2019). Moving beyond generic formulations, the integration of high-throughput omics data with machine learning holds considerable promise for the rational design of personalized culture media. By modeling the metabolic requirements and competitive interactions of community members inferred from metagenomic and metabolomic data, it should be possible to predict media supplements that minimize compositional drift for a given donor. This area remains largely unexplored but offers significant potential for enhancing model personalization.
- **Constructing synthetic consortia:** Ecologically characterized minimal microbiomes, assembled to capture key functional guilds (e.g. primary degraders, cross-feeders, and butyrate producers), offer a complementary approach for mechanistic studies requiring enhanced experimental control. While these simplified systems sacrifice some ecological complexity, they offer superior experimental control and reproducibility for mechanistic and hypothesis-driven studies (Vrancken et al. 2019). Analogous strategies in mouse models, such as the Oligo-Mouse-Microbiota (OMM12) consortium, a defined community of 12 bacterial strains that stably colonizes germ-free mice, have been instrumental in dissecting host–microbiome interactions with high precision and should be considered as important benchmarks when validating *in vitro* minimal consortia (Brugiroux et al. 2016). Ultimately, the integration of well-preserved complex communities for discovery science and defined consortia for mechanism-driven validation will create a complementary ecosystem of *in vitro* tools with distinct but synergistic roles.

### How can we bridge the gap from *in vitro* models to *in vivo* systems and ultimately to clinical applications?

Current translational pipelines typically progress from *in vitro* mechanistic studies to *in vivo* validation and finally to clinical application. However, this linear path is fraught with limitations, and a critical question is whether advanced *in vitro* platforms can reduce, refine, or even replace the *in vivo* step for certain applications. Animal models, particularly murine systems, provide an integrated physiological context that *in vitro* systems cannot yet fully reproduce. Nevertheless, animal models also present well-recognized shortcomings for microbiome science, including host-specific microbial ecology, differences in gut anatomy and transit, immune system divergence from humans, and confounding effects from animal housing (Arrieta et al. 2016). These factors can obscure clinical translatability and slow the development of patient-specific interventions.

A more forward-looking strategy is to develop a parallel translational framework in which increasingly complex *in vitro* models directly inform clinical studies, with animal models deployed only for specific questions that genuinely require whole-organism physiology. By reconstructing key human-specific host–microbiome interfaces, such as patient-derived intestinal organoids, immune-competent co-cultures, and dynamic mucosal environments, advanced *in vitro* systems could provide human-relevant efficacy and safety data that bypass the need for animal experimentation in many cases. This shift would not only accelerate the preclinical pipeline but also address the growing regulatory interest in non-animal methodologies.

Achieving this vision requires coordinated progress across several fronts:

- **Increasing biological and architectural complexity to approach *in vivo*-like functionality:** Future models should integrate native or donor-derived human gut microbiota with more physiologically relevant architectures. 3D scaffolds, mucin-alginate matrices, and microfluidic gut-on-a-chip platforms can physically separate luminal and mucosa-associated microbial niches, capturing the spatial organization critical for microbial ecology and host signaling. Co-culture with patient-derived organoids or primary epithelial cells, stromal components, and immune cells (e.g. macrophages, dendritic cells, or T cells) can recapitulate epithelial barrier function, metabolite absorption, and localized host–microbiota immune interactions. For systemic responses, multi-organ chips that link gut, liver, and immune modules are emerging (Ingber 2022, Liang et al. 2026), allowing the study of microbial metabolite metabolism and systemic immune modulation without whole animals. While these systems are technically demanding, they represent a pathway toward *in vitro* platforms that approximate *in vivo* complexity while retaining human genetic and microbial specificity.
- **Refining experimental design for direct clinical relevance:** Model selection should be purpose-driven, with static systems for high-throughput screening, dynamic bioreactors for longitudinal community ecology, and advanced co-culture or organ-on-a-chip models for studying host responses. Systematic inclusion of microbiota from multiple human donors is essential to capture inter-individual variability, a dimension often lost in inbred animal colonies. Importantly, experimental designs should mimic clinical scenarios: testing interventions in the presence of a complex human microbiota, using disease-relevant donor samples, and monitoring endpoints that align with clinical biomarkers (Özkan et al. 2024). By benchmarking these *in vitro* systems against human clinical data, researchers can iteratively enhance the predictive power of the models themselves. High-throughput platforms that can run parallel experiments with microbiota from dozens of donors will be critical for stratifying personalized responses and identifying generalizable mechanisms
- **Establishing cross-validation frameworks that link *in vitro* readouts to clinical endpoints:** The translational credibility of any *in vitro* model hinges on its ability to reproduce human clinical outcomes. Systematic comparison of *in vitro* outcomes with longitudinal human cohort data, patient-derived multi-omics signatures, FMT responses, or carefully selected clinical benchmarks will quantify translational fidelity. For instance, if a gut-on-a-chip model exposed to a candidate probiotic produces a metabolite shift that mirrors the serum metabolome changes observed in a clinical trial, confidence in that model rises. Regulatory acceptance of non-animal data will demand rigorous qualification: demonstrating that the *in vitro* system reliably predicts specific clinical endpoints across diverse populations. Cross-validation should also include negative controls to establish model specificity, thereby building an evidence base that supports the replacement of animal studies for specific decision contexts.
- **Integrating multi-omics and computational approaches to simulate *in vivo* and clinical outcomes:** Advanced analytical frameworks, including multi-omics data integration (metagenomics, metaproteomics, metatranscriptomics, and metabolomics), community-scale metabolic modeling, and machine learning, are crucial for distilling complex *in vitro* datasets into clinically actionable hypotheses. Metabolic models such as genome-scale metabolic reconstructions of individual gut bacteria and community-level flux balance analysis can predict metabolic outputs under different conditions and identify which taxa are driving therapeutic or adverse effects. When paired with *in vitro* experimental data, these models can forecast personalized responses (Quinn-Bohmann et al. 2024). In the longer term, the development of personalized digital twins, computational models that integrate an individual’s multi-omics profile with data from their own personalized *in vitro* cultures, could predict therapeutic responses directly, bypassing both animal and early-phase human testing for certain applications. This paradigm would shift the translational path from *in vitro→in vivo→*clinical to *in vitro→in silico→*clinical, with animal models reserved for exceptional cases where whole-system physiology is indispensable. Realizing this vision will require investment in data standards, model reproducibility, and close collaboration between experimentalists, bioinformaticians, and clinicians.

## Conclusion

*In vitro* gut models provide a critical bridge between foundational microbiome research and the realization of microbiome-based personalized medicine. By integrating static, dynamic, and microbe–host interaction platforms, these systems reduce the confounding complexity of *in vivo* studies while enabling high-throughput screening and mechanistic investigations. Although sustaining human gut microbiota *ex vivo* remains technically challenging, advances in biomimetic engineering and computational modeling are steadily improving physiological relevance. With continued refinement, *in vitro* gut models are poised to become indispensable tools for unlocking the therapeutic potential of the gut microbiome in precision medicine
